# A new prediction model of hepatocellular carcinoma based on N7-methylguanosine modification

**DOI:** 10.1186/s12876-023-02757-9

**Published:** 2023-04-20

**Authors:** Li Yang, Yi-ran Wang, Zhi-qiang Mou, Ping-fu Xiong, Kun Deng, Jian Wen, Jing Li

**Affiliations:** 1 Department of General Surgery (Hepatobiliary Surgery), The Affiliated Hospital of Southwest Medical University, Luzhou, 646000 China; 2 Academician (Expert) Workstation of Sichuan Province, Luzhou, 646000 China; 3 Nuclear Medicine and Molecular Imaging Key Laboratory of Sichuan Province, Luzhou, 646000 China; 4 Department of General Surgery (Gastrointestinal Surgery), The Affiliated Hospital of Southwest Medical University, Luzhou, 646000 China

**Keywords:** 7-methylguanosine (m7G) modification, lncRNAs, Hepatocellular carcinoma, Immune infiltration, Data mining

## Abstract

**Purpose:**

Hepatocellular carcinoma (HCC) is a kind of primary liver cancer. It is a common malignant tumor of digestive system that is difficult to predict the prognosis of patients. As an important epigenetic modification, N7 methyl guanosine (m7G) is indispensable in gene regulation. This regulation may affect the development and occurrence of cancer. However, the prognosis of long non coding RNAs (lncRNAs) in HCC is limited, especially how m7G-related lncRNAs regulate the development of HCC has not been reported.

**Methods:**

The Cancer Genome Atlas (TCGA) provides us with the expression data and corresponding clinical information of HCC patients we need. We used a series of statistical methods to screen four kinds of m7G-related lncRNAs related to HCC prognosis and through a series of verifications, the results were in line with our expectations. Finally, we also explored the IC50 difference and correlation analysis of various common chemotherapy drugs.

**Result:**

Our study identified four differentially expressed m7g-related lncRNAs associated with HCC prognosis. Survival curve analysis showed that high risk lncRNAs would lead to poor prognosis of HCC patients. M7G signature's AUC was 0.789, which shows that the prognosis model we studied has certain significance in predicting the prognosis of HCC patients. Moreover, our study found that different risk groups have different immune and tumor related pathways through gene set enrichment analysis. In addition, many immune cell functions are significantly different among different risk groups, such as T cell functions, including coordination of type I INF response and coordination of type II INF response. The expression of PDCD1, HHLA2, CTLA-4 and many other immune checkpoints in different risk groups is also different. Additionally, we analyzed the differences of IC50 and risk correlation of 15 chemotherapeutic drugs among different risk groups.

**Conclusion:**

A novel lncRNAs associated with m7G predicts the prognosis of HCC.

**Supplementary Information:**

The online version contains supplementary material available at 10.1186/s12876-023-02757-9.

## Introduction

HCC is one of the most common cancers worldwide, with the highest mortality rate among primary liver cancer [1, 2]. Although curable therapies are available for early-stage HCC, the majority of patients have progressed to advanced HCC at the time of diagnosis [3]. Surgical resection is often the only option for patients with advanced HCC.One of the recommended treatments for unresectable intermediate HCC is transarterial chemoembolization (TACE) [4, 5]. However, TACE cannot completely cure HCC. Drug‑eluting bead (DEB)-TACE, which is considered to be more effective and safer, was also found to have no significant difference with conventional TACE [6]. With the progress of medicine, the overall survival rate (OS) of HCC patients is increasing, but the prognosis prediction and detailed molecular mechanism of HCC are still unclear, which needs to be further clarified [7]. So, it is imperative to find new and effective prognostic markers for HCC. Presently, different modifications of RNA have been found to have more than 160 types [8]. RNA modifications play an important role in gene expression regulation [9]. It has been reported that RNA modification can affect RNA processing, transport, stability and mRNA translation [10]. Therefore, RNA modification plays a key role in developing different diseases, including HCC [11].

N7-methylguanosine (m7G) is an essential epigenetic modification, playing a vital role in gene expression regulation [12]. The methylation of guanosine (G) at N7 position is called m7G, which exists in mRNA 5′cap [13], rRNA [14], tRNA [13], and internal mRNA regions [15]. It is not difficult to see that this modification plays a crucial role in regulating RNA processing, function and metabolism. Research shows that many diseases are related to this modification [16]. For example, WDR4 (WD Repeat Domain 4) is a human methyltransferase complex. Its mutation can lead to facial malformation and even seizures [16]. Previous studies have reported that the degree of neural differentiation is affected by the expression of WDR4 [17]. At the same time, the researchers found that the m7G tRNA methyltransferase METTL1 (methyltransferase like 1) is related to the growth of cancer cells [18]. It is reported that LncRNA is a subset of about 200 nt RNA molecules that regulate gene expression [19]. The researchers found that autophagy-related lncRNAs significantly affect the prognoses of patients with colon adenocarcinoma [20]. An another study found that the fatty aldehyde dehydrogenase (FALDH) gene,ALDH3A2 could effectively predict the prognosis of gastric carcinoma (GC) patients and may be an independent prognostic biomarker [21]. To sum up, we found that lncRNAs are also involved in gene regulation, tumorigenesis, disease development and metastasis [22]. This provides a new way for us to predict tumor prognosis and find new therapeutic targets through bioinformatics analysis. But, the study of m7G regulator modifying lncRNAs to affect tumor development is limited [23], especially in HCC. Therefore, determining that m7G related lncRNAs are as part of the process of HCC will help to determine effective and targeted treatment. This also makes our research very meaningful.

## Materials and methods

### Data sources

Obtain transcriptome information and clinical information of HCC from TCGA database(http://cancergenome.nih.gov/). In the subsequent analysis steps, patients lacking survival information were excluded. Detailed transcriptome data were downloaded from TCGA-LICH queue, including 374 HCC tissues and 50 precancerous tissues. First, hundreds of lnRNAs relating to 29 acknowledged m7G-related genes were selected. In this study m7G-related genes included are shown in Supplementary Table S1 [24]. In our study, we used average probe strength to show the expression level of lncRNA/genes with different probes.

### Screening of differential genes

We use "limma" (R package) to identify m7G-related genes (m7GG) differentially expressed between cancer and normal tissues [25]. The definition of DE-m7GGs (differentially expressed m7GGs) is that the adjusted | log2FC |> 0 and p value < 0.01 are considered as differentially expressed m7GGs. (Supplementary Table S2). In order to study the accuracy, we selected differentially expressed genes according to ap-values < 0.01 and |log2 FC|> 1, they include NUDT10, NCBP2, EIF3D, LARP1, AGO2, NUDT11.Presently, two databases, including WikiPathways and BioPlanet, are considered origins of pathway classification to specify the shared pathways among DE-m7GGs.

### Identification of m7G-related lnRNAs

Our study evaluated the relationship between m7G related LncRNA and HCC using Pearson correlation analysis. The condition of significant correlation is that when *p* < 0.001, the correlation coefficient | R2 |> 0.4. AND four hundred and sixty-one m7G-related lncRNAs (Supplementary Table S3).

### Construction of prognosis model of m7G signature

First, the TCGA-LICH dataset was divided into three parts, one as the joint set, one as the test set, and one as the training set. In our study, we first conducted a single factor analysis of m7G-related lncRNAs. After univariate Cox analysis, the lncRNA with *p* < 0.05 was screened, and then further analyzed by the least absolute contraction and selection operator (LASSO). Finally, we determined the risk lncRNAs in the model through multivariate Cox. Finally, the risk scores of the patients we studied were calculated as follows: (Coefficient TMCC1-AS1 × expression of TMCC1-AS1) + (Coefficient ZNF232-AS1 × expression of ZNF232-AS1) + (Coefficient AL031985.3 × expression of AL031985.3) + (Coefficient MKLN1-AS × expression of MKLN1-AS). If the risk score is greater than the median number, we call it high- risk group; otherwise, it is called low-risk group. We used R package "survival" to evaluate the survival rate between different risk groups [26]. In order to verify whether the risk score of the model is meaningful, we also used the R package "Survival ROC" to analyze receiver operating characteristics (ROC) [27].

### Analysis combined with clinical related information

We obtained the clinicopathological information of each HCC patient from TCGA to analyze the correlation between the clinical information of HCC and the prognosis model we studied. Then, chi square test was used to evaluate the difference of clinicopathological characteristics between different risk groups. Wilcoxon test is also an important test method selected by us. We use it for analysis to evaluate the difference of risk scores in different groups according to clinical pathological characteristics.

### Nomogram analysis

Our study used R package “rms” to analyze the 1-year, 3-year and 5-year survival rates of HCC patients, and displayed the results with Nomogram. The calibration curve is then used to assess the difference between the survival rate predicted by the nomogram and the actual survival rate. Calculation of area under ROC curve (AUC) shows that the prediction ability of nomogram is more accurate than other prediction factors.

### Principal component analysis at molecular level

The significance of principal component analysis (PCA) is to show that our model analysis of m7G-related lncRNAs is superior to direct analysis of all genes set, all lncRNAs set and all m7G- elated lncRNAs.

### The predictive ability of m7G signature is superior to other prognostic models

To verify whether our m7G feature is superior to the previously determined HCC model, we compared our HCC features with other features, including 9 lncRNAs features [28], 11 lncRNAs features [29] and 7 lncRNAs features [30]. We also compared the survival curve and AUC value of each prognostic model.

### Analysis of immune correlation in prognosis model

We used six algorithms to study the immune response of people with different risks, and the results were visualized using bubble charts. In addition, to compare the difference of tumor infiltrating immune cell (TIIC) subsets among different risk groups, we analyzed them through ssGSEA and evaluated the immune function of the two groups. Immune checkpoints are available from reported literature. And we applied the currently developed deconvolution algorithm, including MCPcounter [31], TIMER [32], QUANTISEQ [33], XCELL [34], EPIC [35], CIBERSORT and CIBERSORT-ABS [36].

### Analysis of chemosensitivity

We use “pRRophic” (R package) application algorithm to predict chemical drug IC50, which is significant in studying the difference of chemosensitivity between different risk groups [37]. We used box graph and correlation graph to visualize common chemotherapy drugs.

### Validate the prognosis model at the cellular level

In order to further validate our previous analysis, we have carried out validation at the cellular level. We chose to validate ZNF232-AS1 in the prognostic model because it has never been reported, which may provide new research directions. We first used TRIzol to extract total RNA from cell samples. Next, We use QuantiTect reverse transcription kit to reverse transcribe RNA into cDNA. QRT-PCR was used to evaluate the expression of ZNF232-AS1 in different cell lines. We use GAPDH for internal reference and the primer related information is shown in Supplementary Table S4. Human HCC cells, including SK HEP-1, Huh7, HEPG2, HCCLM3,PLC/PRF/5.and the normal human liver cell line L02 were purchased from the cell bank of the Type Culture Collection of the Chinese Academy of Sciences (Shanghai, China).

### Statistical analysis

R software is used for data analysis. The ap value of both sides is 0.05, which is considered to be statistically significant.

## Results

### Differential expression of m7G-related genes

**Fig. 1:**
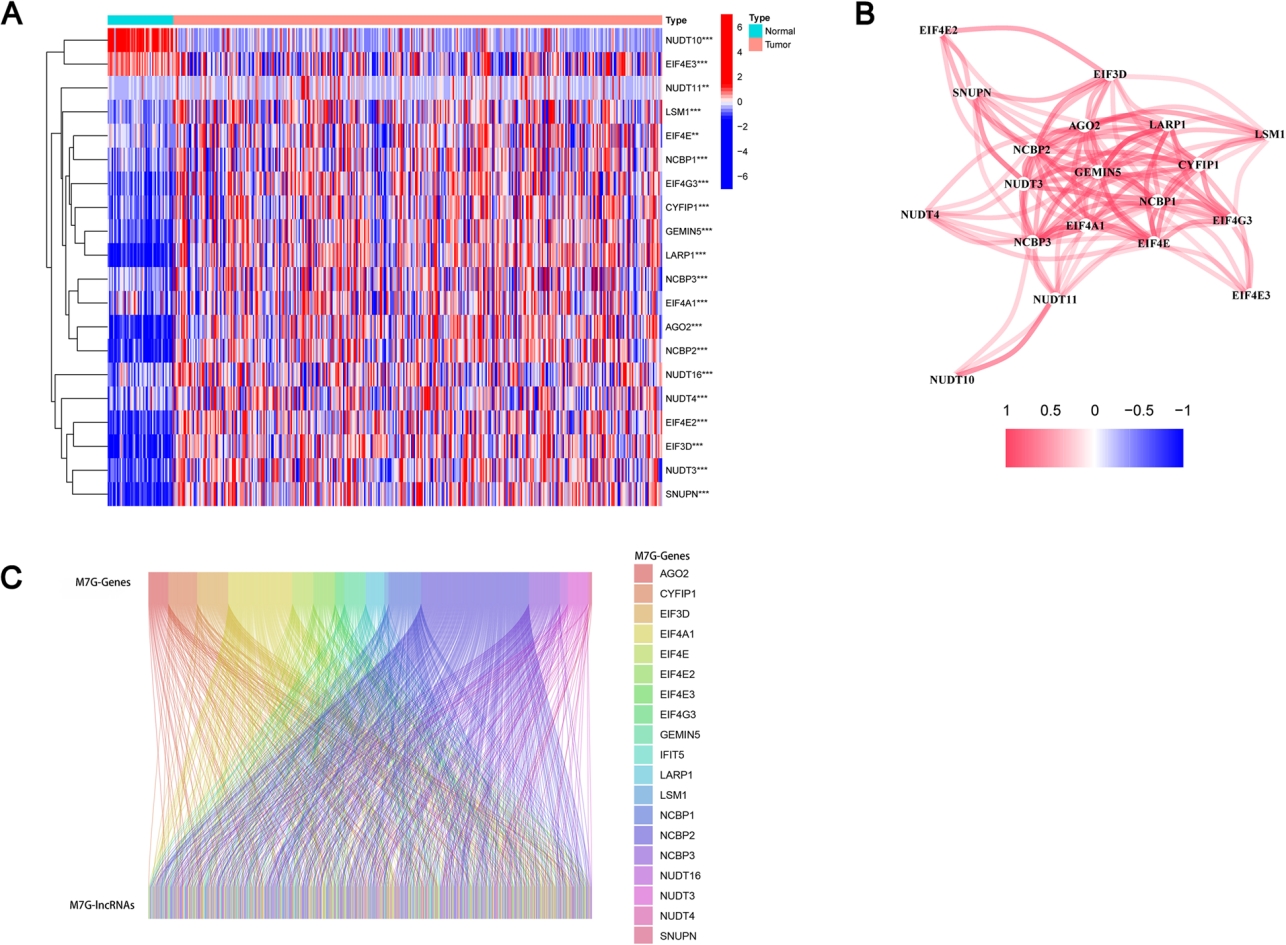
Determine m7G related lncRNAs. **A** Heat map of DE-m7GGs. **B** Correlation network of DE-m7GGs (red represents positive correlations). **C **Sankey diagram shows correlations between m7G regulators and lncRNAS

M7G-related genes were compared in normal and HCC tissues. DEGs are visualized by heat map. (| log2(fold change) |≥ 0 and p value < 0.05) (Fig. 1). See Fig. 1B for co-expression of differential genes. Secondly, we explored lncRNAs co-expressing differentially expressed genes (Fig. 1C). We have concluded that m7g-related genes are almost differentially expressed in HCC and normal tissues. In order to study the accuracy, we selected DEGs based on adjusted p-values < 0.01 and |log2 FC|> 1, they include NUDT10, NCBP2, EIF3D, LARP1, AGO2, NUDT11.

### Establishment of m7G signature

**Fig. 2:**
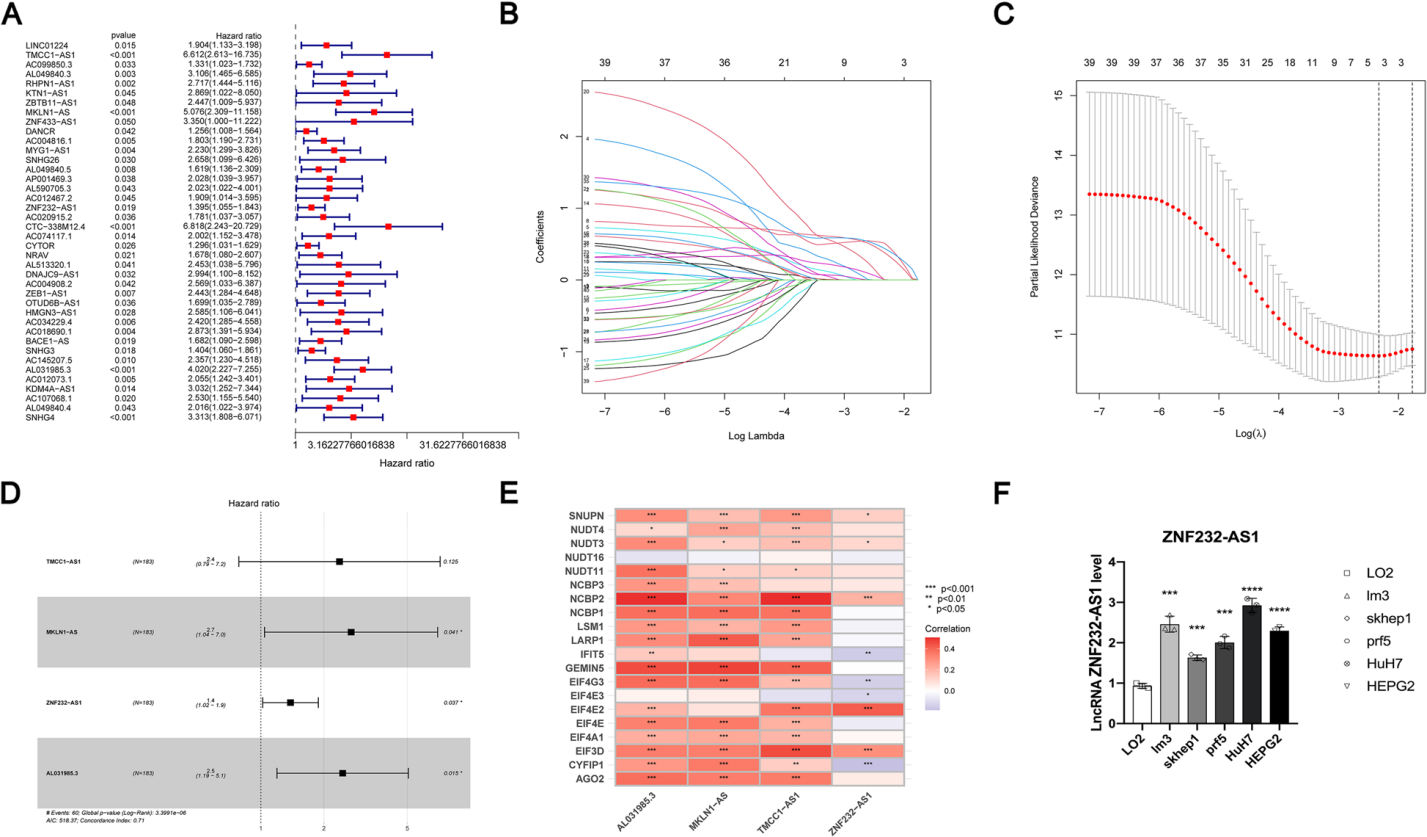
**A** univariate Cox regression analysis showed that 40 m7G-related lncRNAs were associated with the survival of HCC patients. **B**, **C** Lasso Cox regression models identified 4 m7G-related lncRNAs. **D** Four lncRNAs were analyzed by multivariate Cox regression. **E** The co-expression of these four lnRNAs with m7g-related genes. **F** The expression difference of ZNF232-AS1 in the model between liver cancer cell line (lm-3, skhep1, prf5, Huh7, HEPG2) and normal liver cell line (LO-2). *P < 0.05, **P < 0.01, ***P < 0.001, ****P < 0.0001

In conclusion, the entire TCGA cohort contains 375 HCC patients (*n* = 375). Our study randomly divided them into a training cohort containing 183 HCC patients (*n* = 183) and an internal validation cohort containing 182 HCC patients (*n* = 282). Their clinical information is shown in Table 1. Our study found that 40 m7G related lncRNA was contacted with the prognosis of HCC patients, which was obtained through univariate Cox regression analysis (*p* < 0.05) (Fig. 2A). They were further screened by LASSO regression analysis and multivariate Cox analysis and 4 lncRNAs were screened for inclusion in the prognosis model (Fig. 2B-D). Finally, we explored the co-expression of these four lnRNAs with m7G-related genes (Fig. 2E). The risk score was calculated by the expression level of these four m7G related lncRNAs and Cox regression coefficient. Among them, in order to study the difference of ZNF232-AS1 expression between normal liver cell line (LO-2) and liver cancer cell line (lm-3, skhep1, prf5, Huh7, HEPG2), we analyzed it by qRT-PCR (Fig. 2F). Because ZNF232-AS1 has never been reported in previous studies, we found that the results are significant. Therefore, we conducted the following research.

**Table 1:** Clinical information of HCC patients in each cohort

Covariates	Type	Total	Test	Train	*P *value
Age	< = 65	227 (62.19%)	108 (59.34%)	119 (65.03%)	0.3114
Age	> 65	138 (37.81%)	74 (40.66%)	64 (34.97%)	
Gender	FEMALE	119 (32.6%)	58 (31.87%)	61 (33.33%)	0.8517
Gender	MALE	246 (67.4%)	124 (68.13%)	122 (66.67%)	
Grade	G1	55 (15.07%)	27 (14.84%)	28 (15.3%)	0.5333
Grade	G2	175 (47.95%)	81 (44.51%)	94 (51.37%)	
Grade	G3	118 (32.33%)	65 (35.71%)	53 (28.96%)	
Grade	G4	12 (3.29%)	6 (3.3%)	6 (3.28%)	
Grade	unknown	5 (1.37%)	3 (1.65%)	2 (1.09%)	
Stage	Stage I	170 (46.58%)	89 (48.9%)	81 (44.26%)	0.3657
Stage	Stage II	84 (23.01%)	36 (19.78%)	48 (26.23%)	
Stage	Stage III	83 (22.74%)	43 (23.63%)	40 (21.86%)	
Stage	Stage IV	4 (1.1%)	1 (0.55%)	3 (1.64%)	
Stage	unknown	24 (6.58%)	13 (7.14%)	11 (6.01%)	
T	T1	180 (49.32%)	92 (50.55%)	88 (48.09%)	0.619
T	T2	91 (24.93%)	42 (23.08%)	49 (26.78%)	
T	T3	78 (21.37%)	42 (23.08%)	36 (19.67%)	
T	T4	13 (3.56%)	5 (2.75%)	8 (4.37%)	
T	unknown	3 (0.82%)	1 (0.55%)	2 (1.09%)	
M	M0	263 (72.05%)	126 (69.23%)	137 (74.86%)	0.3655
M	M1	3 (0.82%)	1 (0.55%)	2 (1.09%)	
M	unknown	99 (27.12%)	55 (30.22%)	44 (24.04%)	
N	N0	248 (67.95%)	122 (67.03%)	126 (68.85%)	0.5265
N	N1	4 (1.1%)	1 (0.55%)	3 (1.64%)	
N	unknown	113 (30.96%)	59 (32.42%)	54 (29.51%)

### Survival results and multivariate examination

**Fig. 3:**
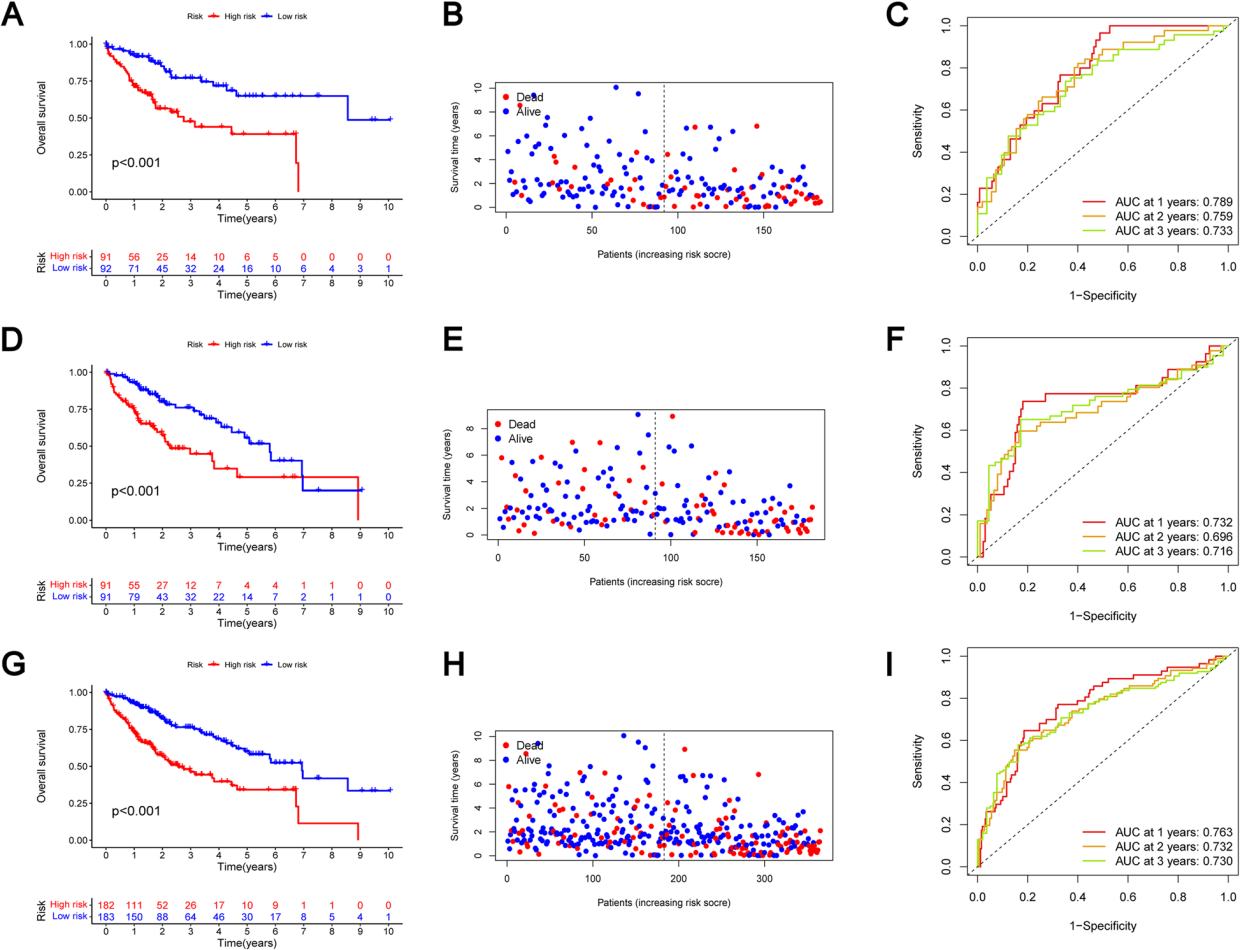
**A** In the training cohort, Kaplan–Meier survival curve of the m7G signature. **B** In the training cohort. the risk plot of the m7G signature (**C**) In the training cohort, the ROC curve of the m7G signature. **D** In the internal validation cohort, Kaplan–Meier survival curve of the m7G signature. **E** In the internal validation cohort, the risk plot of the m7G signature. **F** In the internal validation cohort, the ROC curve of the m7G signature. **G** In the entire TCGA cohort, Kaplan–Meier survival curve of the m7G signature. **H** In the entire TCGA cohort, the ROC curve of the m7G signature. **I** In the entire TCGA cohort, the ROC curve of the m7G signature

**Fig. 4:**
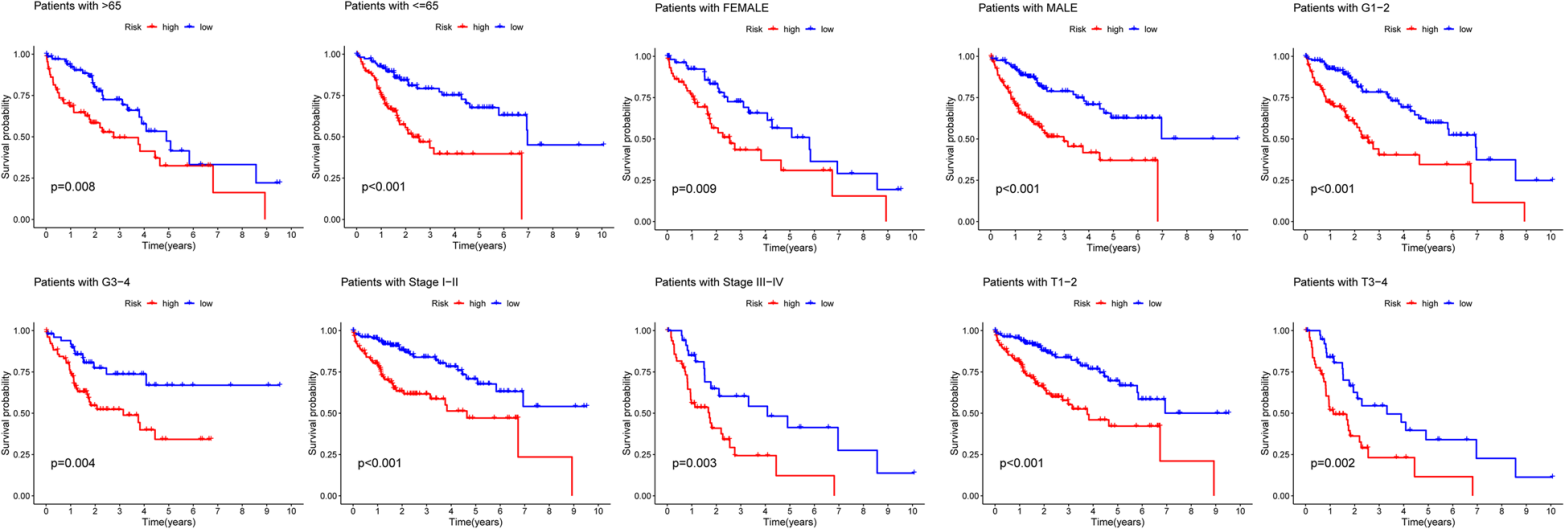
Predictability of prognostic model in different clinicopathological conditions

Our study found that 0.8765 was the median risk score. Next, we will divide the liver cancer patients in the training queue into a high-risk group containing 91 HCC patients (*n* = 91) and a low-risk group containing 92 HCC patients (*n* = 92). Kaplan Meier analysis using log rank test showed that the survival rate of patients in low-risk group was significantly higher than that of patients in high-risk group (Fig. 3A). Similarly, as shown in the risk plot, we found that the mortality of HCC patients was positively correlated with the risk score (Fig. 3B). The time ROC curve showed that 0.789 was the AUC value of 1-year survival period, 0.759 was the AUC value of 2-year survival period and 0.733 was the AUC value of 3-year survival period (Fig. 3C). At the same time, we conducted the same analysis in the entire TCGA cohorts and the internal verification cohorts, and the results are still satisfactory. It is easy to see that through the analysis of the internal training queue, we get the same results as the training set (Fig. 3D-E). In the internal validation cohort, we conducted the same analysis on the internal training queue, and the time ROC curve results showed that 0.732 was the AUC value of 1-year survival, 0.696 was the AUC value of 2-year survival and 0.716 was the AUC value of 3-year survival (Fig. 3F). For the entire TCGA cohorts, our analysis results are the same as above (Fig. 3G-H). And 0.763 was the AUC value of 1-year survival, 0.732 was the AUC value of 2-year survival and 0.730 was the AUC value of 3-year survival (Fig. 3I). In conclusion, the m7G-related lncRNAs we studied can be used to predict the prognosis of HCC patients. More interestingly, we validated the clinical information by grouping. At the same time, we also studied whether the survival curves of different risk groups in different clinical information groups are meaningful, and the results are the same as those we obtained before (Fig. 4).

### Correlation between prognosis model and clinical pathological parameters of HCC

**Fig. 5:**
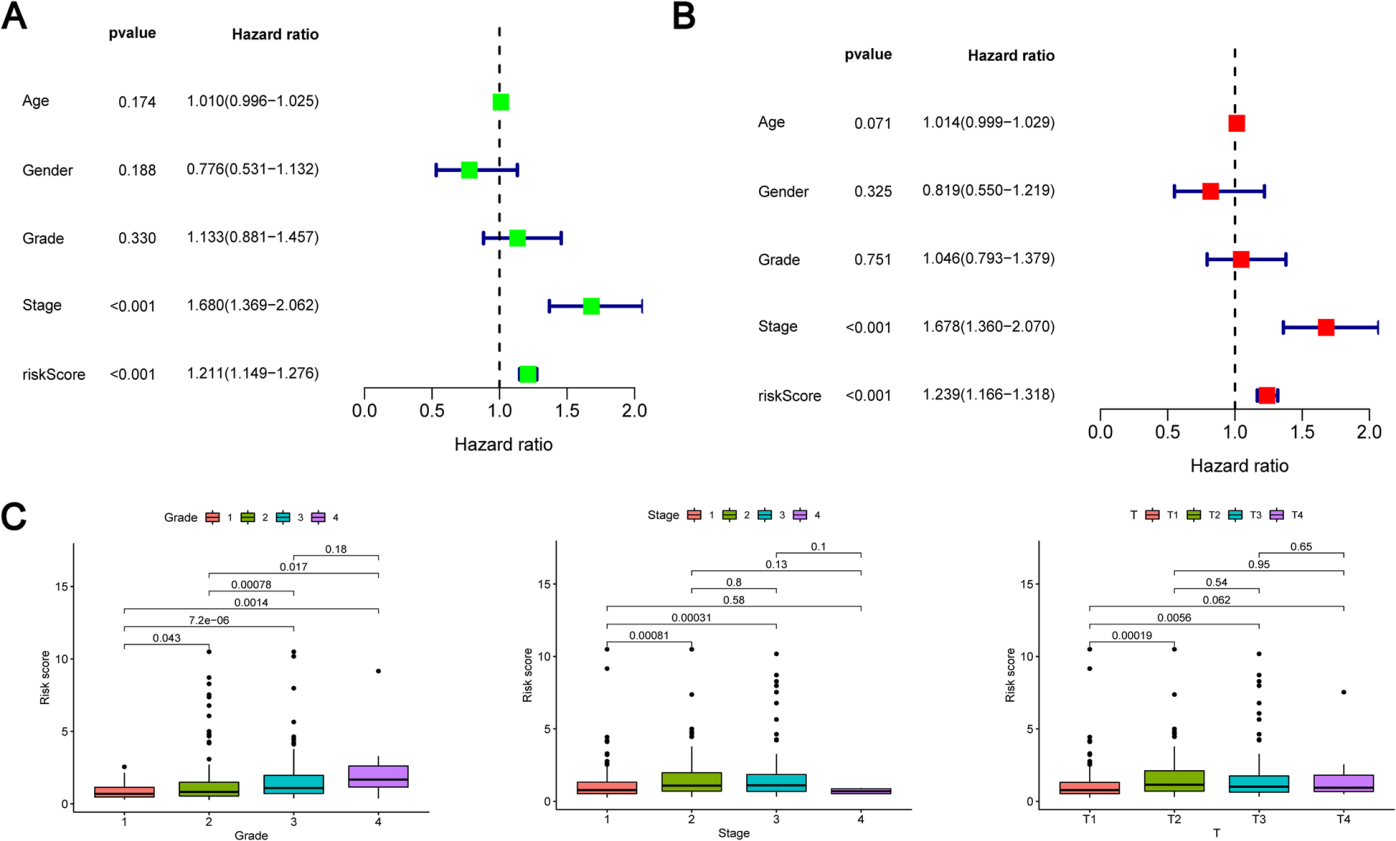
**A** In the training cohort, analysis results of univariate cox regression analysis. **B** In the training cohort, analysis results of multivariate cox regression analysis. **C** Scatterplot shows that high risk score levels are significantly closely related to T classification, tumor grade and pathological stage

Through univariate and multivariate Cox analysis in our study, we found that risk score was independent prognostic factors of OS in HCC patients (Fig. 5A, 5B). More importantly, through our analysis, we found that the risk score was closely related to the grading, T-classification and pathological staging of HCC. We show the more aggressive clinicopathological features by scatter plot (Fig. 5C). These results indicate that the risk score is significantly related to the clinicopathological characteristics of relatively advanced HCC.

### Nomograms for predicting long-term survival of HCC patients

**Fig. 6:**
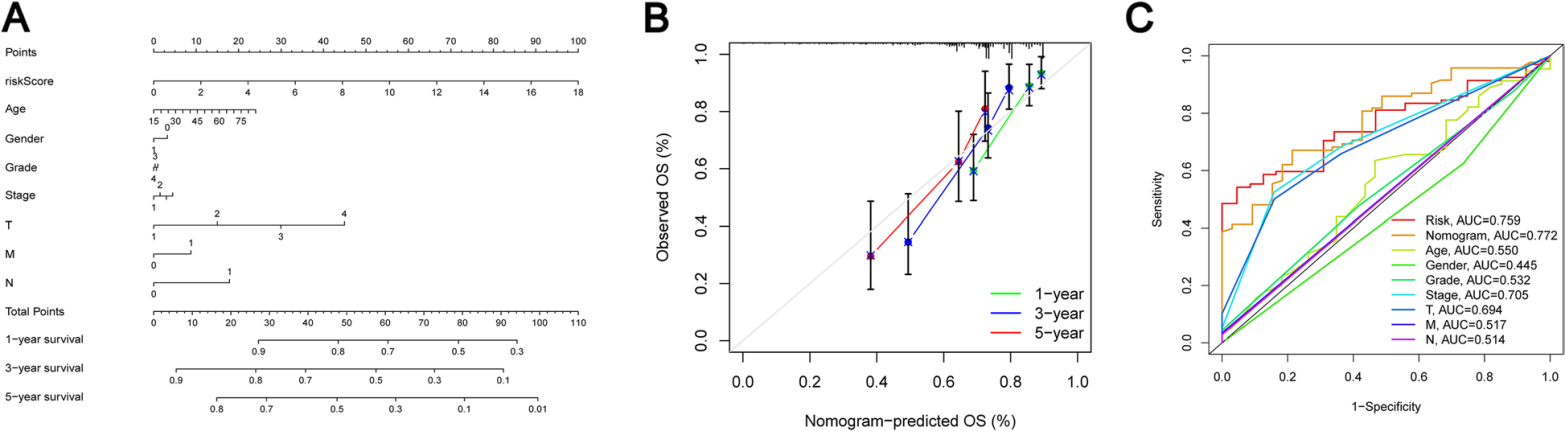
**A** Predictive effect of nomogram on HCC patients. **B** Nomogram calibration map constructed in our study. **C** The AUC value of nomogram and m7G signature was significantly higher than that of other clinical information

To more accurately predict the 1-year, 3-year and 5-year survival rates of patients with HCC patients, we constructed a nomogram of the combined risk score of age, stage and so on (Fig. 6A). Calibration chart shows excellent performance of nomogram (Fig. 6B). The AUC value corresponding to the risk score is 0.759 according to the ROC curve, AUC values corresponding to nomogram was 0.772, AUC values corresponding to age was 0.550, AUC values corresponding to gender was 0.445, AUC values corresponding to tumor grade was 0.532, AUC values corresponding to stage was 0.705, AUC values corresponding to T classification was 0.694, AUC values corresponding to N classification was 0.517 and AUC values corresponding to M classification was 0.514 (Fig. 6C). To sum up, the nomograph we built is very meaningful for predicting the survival prognosis of HCC patients.

### Principal component analysis of m7G signature

**Fig. 7:**
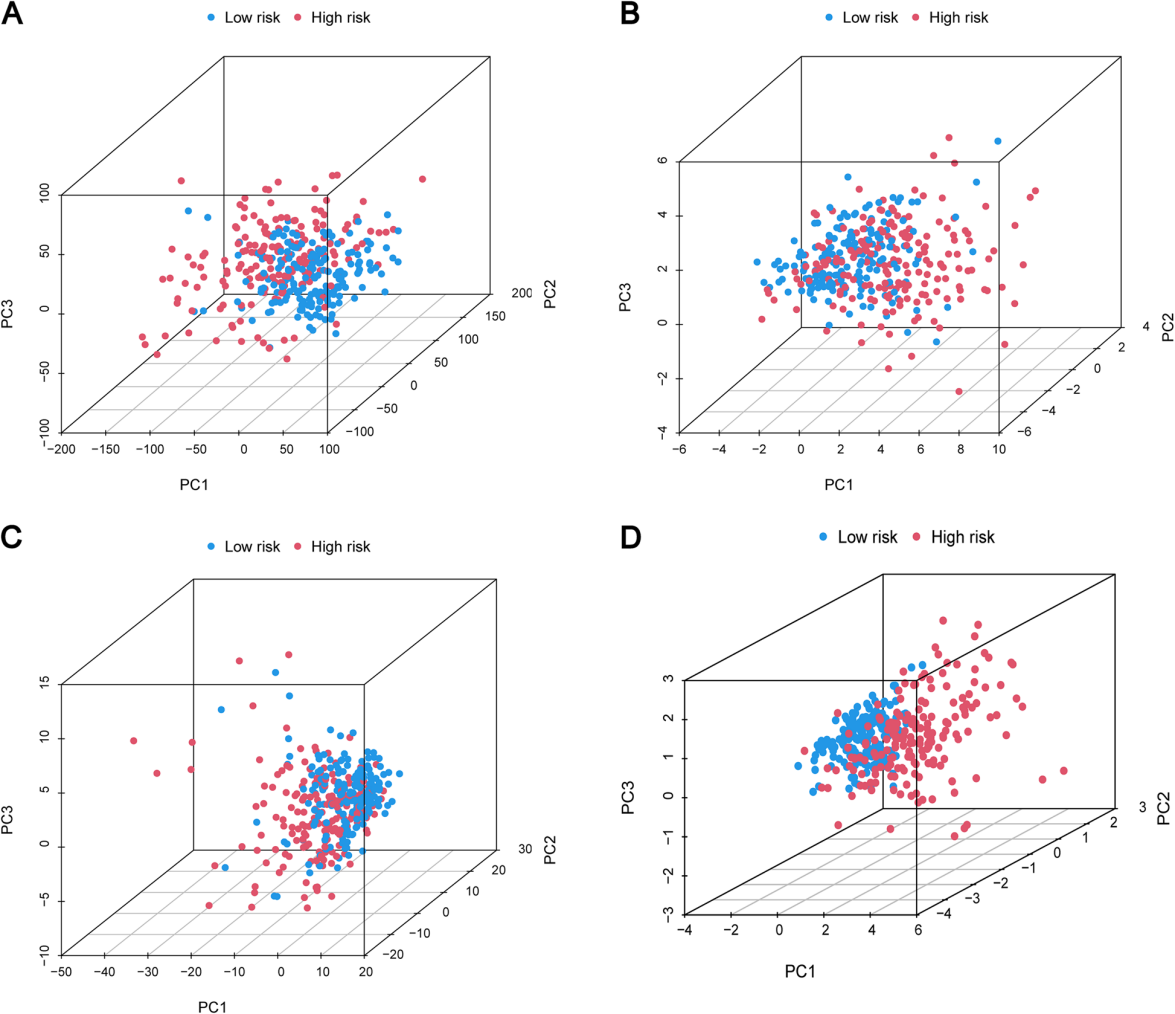
**A** In all genes set, the PCA analysis results. **B** In all m7G-related lncRNAs set, the PCA analysis results. **C** PCA analysis results of all lncRNAs set. **D** PCA analysis results of m7G-related lncRNAs set

For different expression profiles, we used PCA analysis to study different distribution patterns among different risk groups. The low-risk group is represented by blue dots and high-risk group is represented by red dots. We divided four m7G-related lncRNAs set, all genes set, all lncRNAs set, and all m7G-related lncRNAs set into two parts. The results showed that four m7G-related lncRNAs set had the best results (Fig. 7A-D).

### Function analysis of M7G signature

**Fig. 8:**
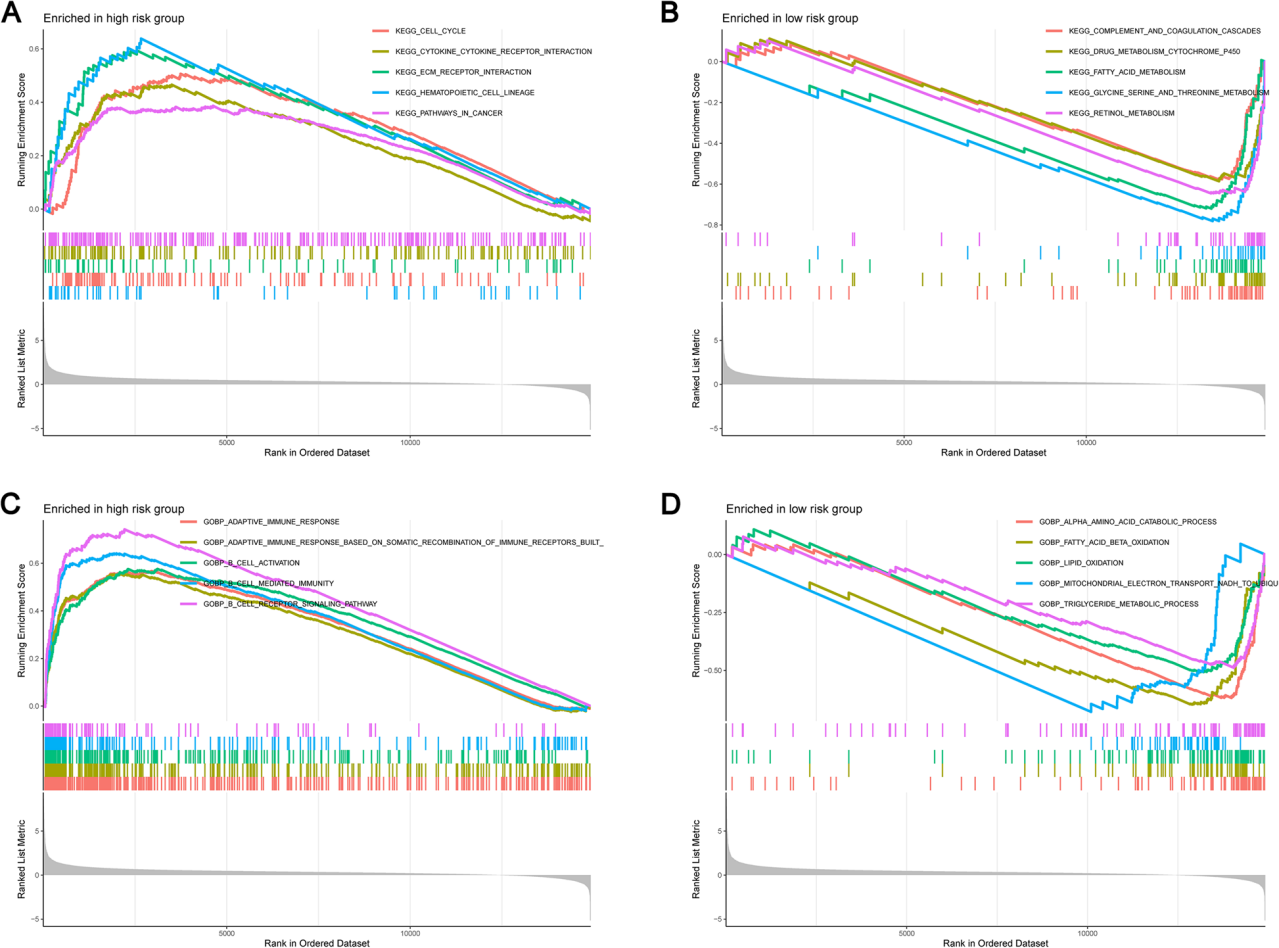
GSEA analysis. **A**-**B** GO analysis results. **C**-**D** KEGG analysis results

In order to study the potential mechanism of these lnRNAs, we conducted enrichment analysis on two different risk populations through Kyoto Encyclopedia of Genes and Genomes (KEGG) and Gene Ontology (GO) and showed the most prominent five functions [38–40]. For m7G signature, KEGG path analysis such as 'cytokine cytokine receptor interaction', 'cell cycle', 'hematopoietic cell lineage', 'ECM receptor interaction', 'pathways in cancer' were mainly concentrated in high-risk groups (Fig. 8A). Relatively low-risk groups, mainly enriched in ' clycine serine and threonine metabolism ', ' metabolism cytochrome P450 ', ' fatty acid metabolism ', ' retinol metabolism ', ' complement and coagulation cascades ' (Fig. 8B). As shown in Fig. 8C, ‘B cell activation’ and ‘adaptive immune response’ were significantly enriched for BPs. More importantly, m7G signature may participate in fatty acids in the oxidation pathway in functional analysis β Oxidation, which further indicates that they may participate in the growth of HCC (Fig. 8D). The above analysis shows that risk score may be related to tumor immunity and tumor microenvironment, which is very important for further study of HCC.

### M7G signature are obviously superior to other prediction models

**Fig. 9:**
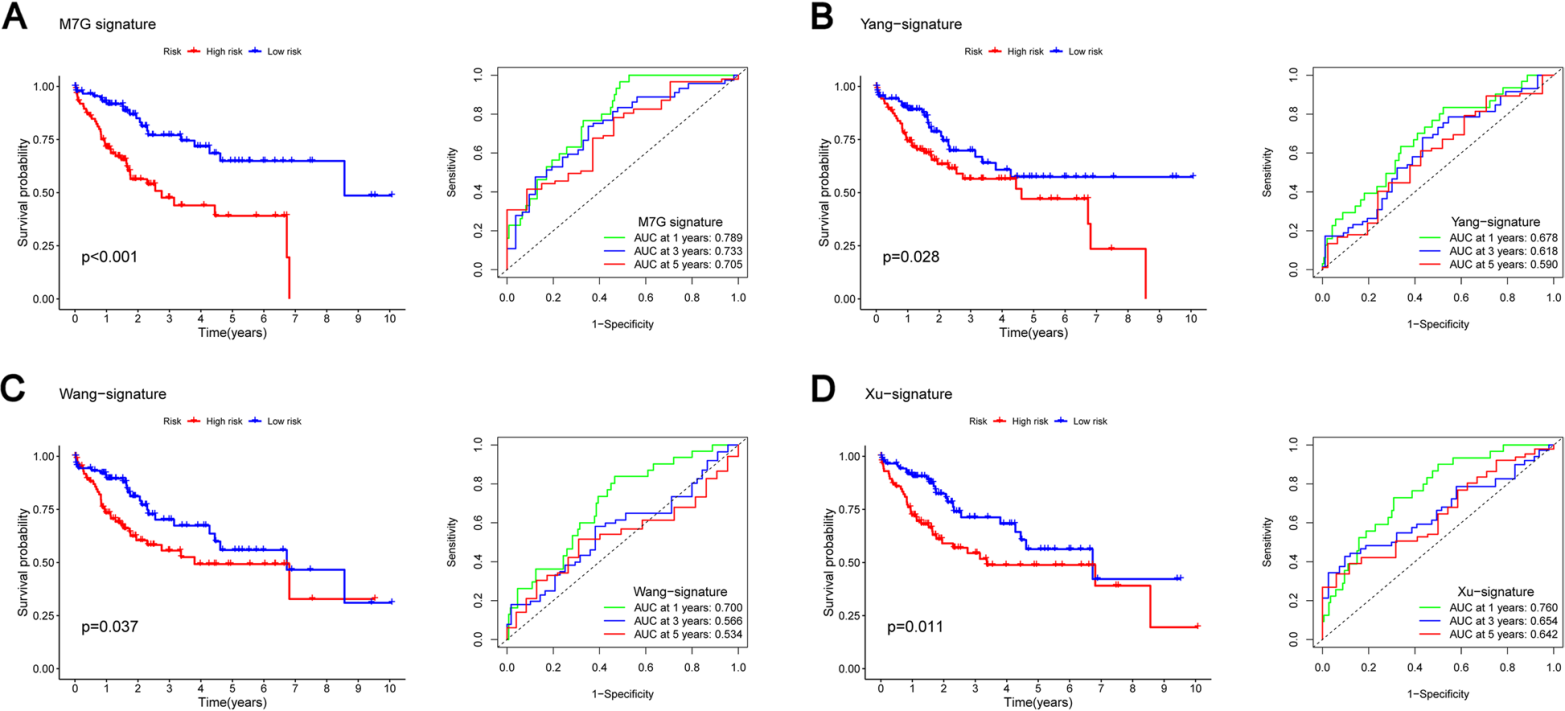
**A** M7G signature’s ROC curve and Kaplan–Meier survival curve. **B**-**D** Yang et al.’s signature, Wang et al.’s signature and Xu et al.’s signature ROC curve and ROC curve Kaplan–Meier survival curve

To verify that the prediction ability of m7G features is the best, we compared m7G signature with some previous prediction models used to predict HCC. As shown in (Fig. 9A-D), the survival curve p value and AUC of our m7G signature are also better than other models. Yang’s signature showed that 0.789 was the AUC value of 1-year survival,0.618 was the AUC value of 3-year and 0.590 was the AUC value of 5-year survival (Fig. 9B). Wang's signature showed that 0.700 was the AUC value of 1-year survival,0.566 was the AUC value of 3-year and 0.534 was the AUC value of 5-year survival (Fig. 9C). Xu’s signature showed that 0.760 was the AUC value of 1-year survival,0.654 was the AUC value of 3-year and 0.643 was the AUC value of 5-year survival (Fig. 9D). The study found that our prognosis model is obviously superior to other prognosis models.

### Correlation analysis for immunity

**Fig. 10:**
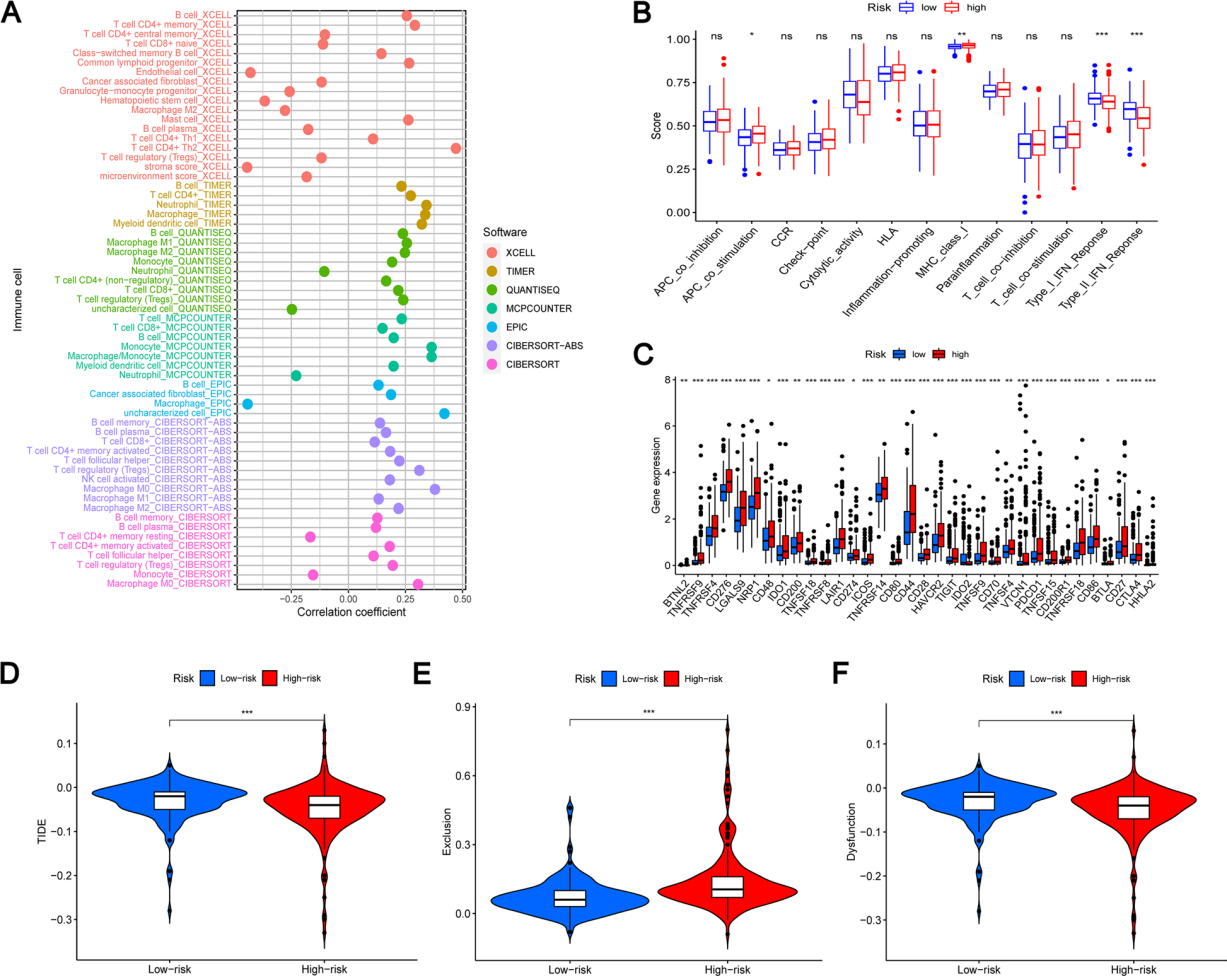
**A** Bubble diagram of immune response of different risk groups based on 7 different algorithms. **B** Analysis of correlation between related functions by ssGSEA and immune cell subsets. **C** Expression of immune checkpoint among different risk groups. **D**-**F** Sensitivity analysis of immunotherapy for different risk population based on TIDE algorithm

In order to compare the immune responses among different risk groups, we used a variety of algorithms, including TIMER, CIBERSORT, CIBERSORT-ABS, QUANTISEQ, MCP, XCELL, and EPIC algorithms. The results are shown in Fig. 10A. Through the correlation analysis between ssGSEA related functions, we found that the coordination of T cell functions was significantly different among patients in different risk groups (Fig. 10B). As we all know, immune checkpoint is very important in cancer treatment, and targeted treatment is the key to immunotherapy. Therefore, we further studied the difference of immune checkpoint expression between different risk groups. Our study found that some meaningful immune checkpoints were highly expressed in high-risk groups, including PDCD1, HHLA2 and CTLA-4 (Fig. 10C). More importantly, the TIDE score, exclusion score and dysfunction score of different risk groups were also significantly different (Fig. 10D-F). Our research results show that the effect of immunotherapy on different risk groups is also significantly different, and high risk may benefit more from immunotherapy. Therefore, our risk model may provide a new way to treat HCC.

### Analysis of differences between different risk groups for chemotherapy

**Fig. 11:**
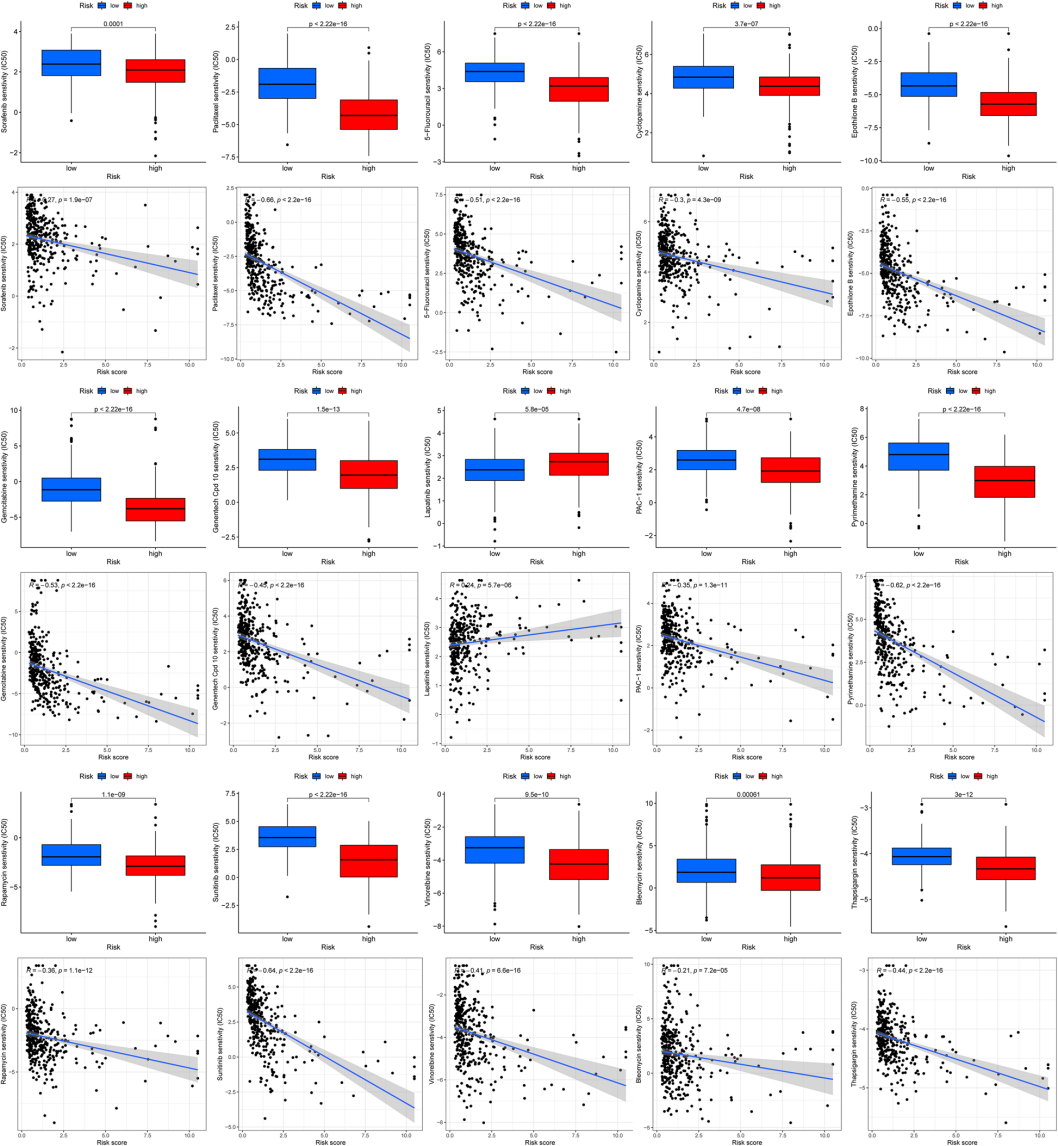
The box chart of IC50 and risk correlation difference of 15 chemotherapy drugs between different risk groups

We discussed the difference and correlation analysis of IC50 of various commonly used chemotherapy drugs among different risk groups. Using the prophetic algorithm, we showed 15 chemotherapeutic drugs with multiple anticancer mechanisms. In addition, the IC50 and correlation difference boxplots of 15 chemotherapy drugs between different risk groups were drawn (Fig. 11).

## Discussion

The research of lncRNAs in tumorigenesis and cancer progression has become a research hotspot Because many studies have shown that lncRNAs are potential diagnostic and prognostic markers of cancer. Therefore, people are more and more enthusiastic about the research of the influence of lncRNA on HCC. This also shows that epigenetic modification is related to the growth of various cancers [41]. In addition, some studies have shown that RNA methylation is related to tumor immunity [42]. However, the pathogenesis of HCC is still unclear and deserves further elucidation. Therefore, we speculate that m7G-related lncRNA also plays an important role in the growth of HCC. It may be possible to explore new treatment schemes. So our research is very meaningful.

First, we obtained HCC data from TCGA, and screened 20 differentially expressed m7G-related genes by comparing the expression of transcriptome data in liver cancer tissues and normal tissues. Then, in order to study the accuracy, we screened six m7G-related genes with significant differential expression through statistical values. In this study, 461 lncRNAs were identified by comparing the expression level of lncRNAs in normal tissues and HCC tissues. Then, based on these 461 differentially expressed lncRNAs, we carried out univariate Cox regression, lasso Cox regression, and multivariate Cox regression analyses, and finally constructed the features containing 4 m7G related lncRNAs. It has previously been reported that three lncRNAs including MKLN1 ‑ AS, AL031985.3 and TMCC1-AS1 in this prognostic model are all related to the growth of HCC. High expression of TMCC1-AS1 in HCC patients may lead to shorter survival [43]. However, the specific mechanism of TMCC1-AS1 on HCC progression has not been studied, which may provide us with a new direction. It was previously reported that MKLN1 ‑ AS can increase the expression level of HDGF, thereby promoting the growth of HCC [44]. Research shows that AL031,985.3 may participate in the process of apoptosis and autophagy to affect the growth of HCC [45–47]. But ZNF232-AS1 in our prognostic model has never been reported. If the next research is aimed at ZNF232-AS1, by studying its mechanism of action, it may bring new hope to HCC patients and provide new choices in the treatment of HCC. Similarly, research on the role of m7G-related lncRNAs in the growth of HCC is still limited. Therefore, the specific impact mechanism of m7G-related lncRNAs on cancer remains unclear. Therefore, it is necessary to find new prognostic markers of lncRNAs, which is also the original intention of this study. These findings of this study may become a gospel for HCC patients in the future.

It is worth mentioning that we have developed four prognosis models of m7G-related lncRNAs (TMCC1-AS1, MKLN1-AS, ZNF232-AS1, and AL031985.3) and all the research results are satisfactory. For example, the response to immunotherapy varies greatly among different risk groups. In order to confirm the reliability and independence of the prognosis model, we have verified many methods and found that the prognosis model we studied is not only feasible but also practical.

During liver injury, the changes of liver microenvironment are mainly characterized by the imbalance of extracellular matrix (ECM), which will promote the growth of HCC [48]. And the analysis of lncRNAs in the prognosis model by KEGG, we found that these lncRNAs participated in ECM receiver interaction in high-risk groups It shows that these lncRNAs are indeed closely related to HCC. More interestingly, we analyzed the immune related functions of different risk groups through GO, and the results showed that patients in high-risk groups were closely related to immune regulation. Previous studies have shown that immunity also plays an important role in the prognosis of other digestive system tumors [49]. This indicates that the intervention aimed at immunity may affect the growth of tumors. Therefore, we boldly speculate that the low-risk group promotes tumor progression through immunosuppression or immune escape. We analyzed the difference of immune microenvironment between different risk groups to verify our conjecture. Importantly, in the past few years, immunotherapy has become the most important means of anti-cancer. In addition, In our study, we found that most TIIC cells were positively related to the risk score, such as macrophages. Then, we focused on the functional differences of T cells between different risk groups. Through further analysis of different risk groups, we also found that their coordination functions in T-cell type I INF response and type II INF response were different. As we all know, targeting immune checkpoints to treat cancer has become a new anti-cancer method. Studies have shown that the evaluation of the effect of immunotherapy can be reflected by the expression of immune checkpoints [50]. Therefore, we further studied the correlation between M7G signature and the expression of immune checkpoints biomarkers, and studied the expression level of immune checkpoints biomarkers in HCC patients of different risk groups. Nowadays, immunotherapy is very important for patients with advanced HCC. At the same time, studies have shown that immunotherapy with anti PDCD1 and anti CTLA-4 antibodies is effective in the treatment of advanced liver cancer [51]. Studies have shown that inducing M2 polarization and chemotaxis of macrophages in HCC can promote immune escape of HCC [52]. At the same time, NRP1 will be highly expressed in HCC tissues and cell lines, and inhibiting the expression of NRP1 will slow down the development of HCC [53]. However, our research further found that low-risk groups immunization had a greater chance of escaping. To sum up, the M7G signature we have studied can predict the immune characteristics of HCC. Our analysis results show that high-risk people are more sensitive to immunotherapy. Finally, we studied the difference and correlation analysis of IC50 of various commonly used chemotherapy drugs among different risk groups. Importantly, our study found that the effect of sorafenib, a first-line chemotherapy drug for HCC, was significantly different risk groups. Through our research and analysis, we found that high-risk groups are more likely to benefit from chemotherapy.

Inevitably, this study has some limitations. First, the data of this study are all from TCGA, the data used for the analysis may not be fully applicable to patients in a particular region because of differences in genetics between races. There may also be errors and bias caused by the inherent defects of the TCGA, such as insufficient clinical follow-up time, missing clinical data, and data processing by multiple institutions. In the future, we may integrate multiple databases to obtain more clinical samples for data mining. Secondly, there may be statistical differences caused by prognostic analysis software and algorithm, and the validation method of prognostic model is relatively simple, and its molecular mechanism still needs to be further verified by basic experiments. Third, there are still several gaps in the study of m7G-related lncRNAs. Our next work needs to explore the specific mechanism of m7G related lncRNA affecting HCC, in order to further prove its potential as a new therapeutic target for HCC, which we believe is a very meaningful study.

## Conclusion

In short, we found a new risk model to predict the prognosis of HCC patients, which includes four m7G-related lncRNAs. Through our research, we found that this prognostic model has important value in predicting the survival rate of HCC patients. Targeting m7G-related lncRNAs to treat HCC may bring good news to HCC patients.

## Supplementary Information

**Additional file 1: Table S1.** M7G related genes used in this study.

**Additional file 2: Table S2.** The differentially expressed m7G-related genes between HCC and normal samples.

**Additional file 3: Table S3.** 461 m7G-related lncRNAs.

**Additional file 4: Table S4.** Premier sequences for qRT‒PCR Analysis.

## Data Availability

All data and materials used in this study are available in TCGA (http://cancergenome.nih.gov/).

## Abbreviations

HCC: Hepatocellular carcinoma
M7G: N7 methyl guanosine
LncRNA: Long non-coding RNA
TCGA: The Cancer Genome Atlas
ROC: Receiver Operating Characteristic
PCA: Principal Component Analysis
TIIC: Tumor Infiltrating Immune Cell
GO: Gene Ontology
KEGG: Kyoto Encyclopedia Of Genes And Genomes
OS: Overall Survival
WDR4: WD Repeat Domain 4
METTL1: Methyltransferase Like 1
DEG: Differentially Expressed Gene
AUC: Area Under Curve
PDCD1: Recombinant Protein Of Programmed Cell Death 1
CTLA-4: Cytotoxic T Lymphocyte-associated Antigen-4
AJCC: American Joint Committee on Cancer
ECM: Extracellular Matrix
HOTAIR: HOX Transcript Antisense Intergenic RNA
UCA1: Urothelial Carcinoma Associated 1
CCAT1: Colon Cancer Associated Transcript
HDGF: Hepatoma-derived growth factor
NRP1: Neuropilin-1
GSEA: Gene Set Enrichment Analysis
IC50: Half Maximal Inhibitory Concentration

## References

[1] Ruan Q, Wang H, Burke LJ, Bridle KR, Li X, Zhao CX (2020). Therapeutic modulators of hepatic stellate cells for hepatocellular carcinoma. Int J Cancer.

[2] Morse MA, Sun W, Kim R, He AR, Abada PB, Mynderse M (2019). The role of angiogenesis in hepatocellular carcinoma. Clin Cancer Res.

[3] Forner A, Llovet JM, Bruix J (2012). Hepatocellular carcinoma. Lancet.

[4] Heimbach JK, Kulik LM, Finn RS, Sirlin CB, Abecassis MM, Roberts LR (2018). AASLD guidelines for the treatment of hepatocellular carcinoma. Hepatology.

[5] Bruix J, Sherman M, American Association for the Study of Liver D (2011). Management of hepatocellular carcinoma: an update. Hepatology.

[6] Wang H, Cao C, Wei X, Shen K, Shu Y, Wan X (2020). A comparison between drug-eluting bead-transarterial chemoembolization and conventional transarterial chemoembolization in patients with hepatocellular carcinoma: A meta-analysis of six randomized controlled trials. J Cancer Res Ther.

[7] Marquardt JU, Galle PR, Teufel A (2012). Molecular diagnosis and therapy of hepatocellular carcinoma (HCC): an emerging field for advanced technologies. J Hepatol.

[8] Zhao LY, Song J, Liu Y, Song CX, Yi C (2020). Mapping the epigenetic modifications of DNA and RNA. Protein Cell.

[9] Shi H, Wei J, He C (2019). Where, when, and how: context-dependent functions of RNA methylation writers, readers, and erasers. Mol Cell.

[10] Roost C, Lynch SR, Batista PJ, Qu K, Chang HY, Kool ET (2015). Structure and thermodynamics of N6-methyladenosine in RNA: a spring-loaded base modification. J Am Chem Soc.

[11] Chen Y, Peng C, Chen J, Chen D, Yang B, He B (2019). WTAP facilitates progression of hepatocellular carcinoma via m6A-HuR-dependent epigenetic silencing of ETS1. Mol Cancer.

[12] Dai C, Feng P, Cui L, Su R, Chen W, Wei L (2021). Iterative feature representation algorithm to improve the predictive performance of N7-methylguanosine sites. Brief Bioinform.

[13] Cowling VH (2009). Regulation of mRNA cap methylation. Biochem J.

[14] Guy MP, Phizicky EM (2014). Two-subunit enzymes involved in eukaryotic post-transcriptional tRNA modification. RNA Biol.

[15] Malbec L, Zhang T, Chen YS, Zhang Y, Sun BF, Shi BY (2019). Dynamic methylome of internal mRNA N(7)-methylguanosine and its regulatory role in translation. Cell Res.

[16] Ma J, Zhang L, Chen J, Song B, Zang C, Liu H (2021). m(7)GDisAI: N7-methylguanosine (m(7)G) sites and diseases associations inference based on heterogeneous network. BMC Bioinformatics.

[17] Lin S, Liu Q, Lelyveld VS, Choe J, Szostak JW, Gregory RI (2018). Mettl1/Wdr4-Mediated m(7)G tRNA Methylome Is Required for Normal mRNA Translation and Embryonic Stem Cell Self-Renewal and Differentiation. Mol Cell.

[18] Barbieri I, Tzelepis K, Pandolfini L, Shi J, Millan-Zambrano G, Robson SC (2017). Promoter-bound METTL3 maintains myeloid leukaemia by m(6)A-dependent translation control. Nature.

[19] Zhang T, Li K, Zhang ZL, Gao K, Lv CL (2021). LncRNA Airsci increases the inflammatory response after spinal cord injury in rats through the nuclear factor kappa B signaling pathway. Neural Regen Res.

[20] Wu D, Yin Z, Ji Y, Li L, Li Y, Meng F (2021). Identification of novel autophagy-related lncRNAs associated with a poor prognosis of colon adenocarcinoma through bioinformatics analysis. Sci Rep.

[21] Yin Z, Wu D, Shi J, Wei X, Jin N, Lu X (2020). Identification of ALDH3A2 as a novel prognostic biomarker in gastric adenocarcinoma using integrated bioinformatics analysis. BMC Cancer.

[22] Gupta RA, Shah N, Wang KC, Kim J, Horlings HM, Wong DJ (2010). Long non-coding RNA HOTAIR reprograms chromatin state to promote cancer metastasis. Nature.

[23] Luo Y, Yao Y, Wu P, Zi X, Sun N, He J (2022). The potential role of N(7)-methylguanosine (m7G) in cancer. J Hematol Oncol.

[24] Tomikawa C (2018). 7-Methylguanosine Modifications in Transfer RNA (tRNA). Int J Mol Sci.

[25] Ritchie ME, Phipson B, Wu D, Hu Y, Law CW, Shi W (2015). limma powers differential expression analyses for RNA-sequencing and microarray studies. Nucleic Acids Res.

[26] Rizvi AA, Karaesmen E, Morgan M, Preus L, Wang J, Sovic M (2019). gwasurvivr: an R package for genome-wide survival analysis. Bioinformatics.

[27] Mandrekar JN (2010). Receiver operating characteristic curve in diagnostic test assessment. J Thorac Oncol.

[28] Xu Z, Peng B, Liang Q, Chen X, Cai Y, Zeng S (2021). Construction of a Ferroptosis-Related Nine-lncRNA Signature for Predicting Prognosis and Immune Response in Hepatocellular Carcinoma. Front Immunol.

[29] Wang A, Lei J (2021). Identification of an 11-lncRNA signature with high performance for predicting the prognosis of hepatocellular carcinoma using bioinformatics analysis. Medicine (Baltimore).

[30] Yang S, Zhou Y, Zhang X, Wang L, Fu J, Zhao X (2021). The prognostic value of an autophagy-related lncRNA signature in hepatocellular carcinoma. BMC Bioinformatics.

[31] Dienstmann R, Villacampa G, Sveen A, Mason MJ, Niedzwiecki D, Nesbakken A (2019). Relative contribution of clinicopathological variables, genomic markers, transcriptomic subtyping and microenvironment features for outcome prediction in stage II/III colorectal cancer. Ann Oncol.

[32] Li T, Fan J, Wang B, Traugh N, Chen Q, Liu JS (2017). TIMER: a web server for comprehensive analysis of tumor-infiltrating immune cells. Cancer Res.

[33] Plattner C, Finotello F, Rieder D (2020). Deconvoluting tumor-infiltrating immune cells from RNA-seq data using quanTIseq. Methods Enzymol.

[34] Aran D, Hu Z, Butte AJ (2017). xCell: digitally portraying the tissue cellular heterogeneity landscape. Genome Biol.

[35] Racle J, de Jonge K, Baumgaertner P, Speiser DE, Gfeller D (2017). Simultaneous enumeration of cancer and immune cell types from bulk tumor gene expression data. Elife.

[36] Chen B, Khodadoust MS, Liu CL, Newman AM, Alizadeh AA (2018). Profiling Tumor Infiltrating Immune Cells with CIBERSORT. Methods Mol Biol.

[37] Geeleher P, Cox N, Huang RS (2014). pRRophetic: an R package for prediction of clinical chemotherapeutic response from tumor gene expression levels. PLoS ONE.

[38] Kanehisa M, Furumichi M, Sato Y, Kawashima M, Ishiguro-Watanabe M (2023). KEGG for taxonomy-based analysis of pathways and genomes. Nucleic Acids Res.

[39] Kanehisa M (2019). Toward understanding the origin and evolution of cellular organisms. Protein Sci.

[40] Kanehisa M, Goto S (2000). KEGG: kyoto encyclopedia of genes and genomes. Nucleic Acids Res.

[41] Gu L, Frommel SC, Oakes CC, Simon R, Grupp K, Gerig CY (2015). BAZ2A (TIP5) is involved in epigenetic alterations in prostate cancer and its overexpression predicts disease recurrence. Nat Genet.

[42] Zhang M, Song J, Yuan W, Zhang W, Sun Z (2021). Roles of RNA Methylation on Tumor Immunity and Clinical Implications. Front Immunol.

[43] Chen C, Su N, Li G, Shen Y, Duan X (2021). Long non-coding RNA TMCC1-AS1 predicts poor prognosis and accelerates epithelial-mesenchymal transition in liver cancer. Oncol Lett.

[44] Gao W, Chen X, Chi W, Xue M (2020). Long noncoding RNA MKLN1AS aggravates hepatocellular carcinoma progression by functioning as a molecular sponge for miR6543p, thereby promoting hepatomaderived growth factor expression. Int J Mol Med.

[45] Chen ZA, Tian H, Yao DM, Zhang Y, Feng ZJ, Yang CJ (2021). Identification of a Ferroptosis-Related Signature Model Including mRNAs and lncRNAs for Predicting Prognosis and Immune Activity in Hepatocellular Carcinoma. Front Oncol.

[46] Wu ZH, Li ZW, Yang DL, Liu J (2021). Development and Validation of a Pyroptosis-Related Long Non-coding RNA Signature for Hepatocellular Carcinoma. Front Cell Dev Biol.

[47] Jia Y, Chen Y, Liu J (2020). Prognosis-Predictive Signature and Nomogram Based on Autophagy-Related Long Non-coding RNAs for Hepatocellular Carcinoma. Front Genet.

[48] Mazza G, Telese A, Al-Akkad W, Frenguelli L, Levi A, Marrali M (2019). Cirrhotic Human Liver Extracellular Matrix 3D Scaffolds Promote Smad-Dependent TGF-beta1 Epithelial Mesenchymal Transition. Cells.

[49] Li J, Han T, Wang X, Wang Y, Chen X, Chen W (2022). Construction of a Novel Immune-Related mRNA Signature to Predict the Prognosis and Immune Characteristics of Human Colorectal Cancer. Front Genet.

[50] Bethmann D, Feng Z, Fox BA (2017). Immunoprofiling as a predictor of patient's response to cancer therapy-promises and challenges. Curr Opin Immunol.

[51] Zongyi Y, Xiaowu L (2020). Immunotherapy for hepatocellular carcinoma. Cancer Lett.

[52] Wang R, Guo H, Tang X, Zhang T, Liu Y, Zhang C (2022). Interferon Gamma-Induced Interferon Regulatory Factor 1 Activates Transcription of HHLA2 and Induces Immune Escape of Hepatocellular Carcinoma Cells. Inflammation.

[53] Lin J, Zhang Y, Wu J, Li L, Chen N, Ni P (2018). Neuropilin 1 (NRP1) is a novel tumor marker in hepatocellular carcinoma. Clin Chim Acta.

