# Supplementary material for: A new prediction model of hepatocellular carcinoma based on N7-methylguanosine modification
Source: BMC Gastroenterol. 2023 Apr 20;23:131. doi: 10.1186/s12876-023-02757-9 (PMC10120187; doi:10.1186/s12876-023-02757-9)
Supplement: Supplementary file 4 — Additional file 4: Table S4. Premier sequences for qRT‒PCR Analysis. [file 12876_2023_2757_MOESM4_ESM.docx]

**Table S4: Premier Sequences for qRT‒PCR Analysis**

| Premier | Sequences (5’-3’) |
| --- | --- |

ZNF232-AS1-F GAGATGAAATTGACAGAGCCTG

ZNF232-AS1-R TCATAGTGGAGAAAAGCCCTATG

GAPDH-F CATCATCCCTGCCTCTACTGG

GAPDH-R GTGGGTGTCGCTGTTGAAGTC

|  |  |  |  |  |
| --- | --- | --- | --- | --- |
|  |  |  |  |  |
